# Postoperative swelling: influence of a negative pressure application in comparison to manual lymphatic drainage after total knee arthroplasty—a randomized controlled trial

**DOI:** 10.1007/s00590-025-04313-5

**Published:** 2025-05-18

**Authors:** Maximilian Weber, Johannes Oppermann, Christine Lummer, Klaus Dresing, Sebastian Wegmann, Peer Eysel, Lars Müller, Andreas Harbrecht

**Affiliations:** 1 University Hospital Cologne, Cologne, Germany; 2 GHO - Orthopaedic Trauma Medical Practice Mainz, Mainz, Germany; 3 Universitätsmedizin Göttingen, Göttingen, Germany

**Keywords:** Edema, Rehabilitation, Physiotherapy, TKA, MLD

## Abstract

**Purpose:**

Postoperative soft tissue swelling is a significant factor influencing outcome after total knee arthroplasty (TKA). This prospective study aims to investigate the influence of technical device assisted negative pressure therapy (NP) on early functional outcome after TKA. NP was therefore compared to manual lymphatic drainage (MLD).

**Methods:**

A total of 50 consecutive patients undergoing primary TKA were enrolled. 25 subjects per group were either treated by conventional MLD or NP. Primary outcome parameter was defined as circumference of the affected limb in cm postoperative up to seven days postoperatively. Secondary outcome parameters were: Duration of hospitalization, subjective perception of pain (measured via visual analogue scale, VAS), affected limb’s ROM.

**Results:**

NP showed an overall equivalent influence compared to MLD in reducing lower limb swelling, recovering of mobility and length of hospital stay after TKA. Moreover, the application of NP showed a significant decrease in overall pain perception compared to manual lymphatic drainage on day 2 and forth after surgery (*p* < .05).

**Conclusions:**

Our findings show that NP could be a useful device in clinical routine treating postoperative swelling after TKA. Its application is simple and effective for the patient. Particularly given the shortage of healthcare workers and physical therapists, there is a need for supportive measures that NP could fulfil.

## Introduction

Within last decades, there has been a worldwide increasing number of primary total knee arthroplasty (TKA). Essential factors for this include demographic development, advances in medical care, increasing prevalence of obesity and a high functional physical demand [1–3].

Postoperative soft tissue swelling is considered to be a significant factor influencing outcome after surgical procedures, particularly after TKA [4]. This is mainly caused by a pathophysiological lymphedema due to protein and water retention in the tissue as a result of perioperative damage to lymphatic vessels and a consecutive lymphatic flow disturbance [5–7]. This can significantly affect various parameters such as postoperative mobilization, pain, and subsequently the range of motion (ROM) of the affected knee joint. Furthermore, lymphedema is considered a significant risk factor for numerous postoperative complications, including loosening of prosthetic components, wound healing disorders, wound infections, deep vein thrombosis and pulmonary artery embolism [8].

In order to prevent such complications and optimize the overall functional outcome too, a variety of swelling-reducing measures have been presented in the past, including various applications such as cryotherapy and varying postoperative positioning of the knee joint [9–12].

Generally accepted is currently the rapid mobilization of the affected joint and decongestion of the associated limb, including fast-track programs [13, 14]. Manual lymphatic drainage (MLD) is a well-recognized procedure and is nowadays part of the standardized post-treatment concept after TKA [15–17].

MLD bases on the concept of activating the lymphatic tissue to absorb stagnated fluid from the tissue into the lymphatic system. Accordingly, the fluid is directed to the central lymphatic trunks. The therapist can move protein-rich edema fluid from a swollen area of the body to a healthy area. He hereby treats several regions of the body to activate different areas of the lymphatic system. Due to technological advancements, novel treatment methods are frequently introduced. Particularly in view of the current development of the shortage of skilled workers, innovative and efficient treatment methods that relieve the burden on the individual are of interest. *Lymphatouch®* (LymphaTouch Inc., Valimotie, Helsinki, Finland) is a technical device using negative pressure (NP) to activate the lymphatic drainage channels and reduce swelling by creating negative pressure in the process. The positive effect on reducing postoperative swelling could been shown in the context of surgical procedures following injuries to the upper extremity [18–20].

This prospective study aims to investigate the influence of NP on early functional outcome after TKA. NP was therefore compared to MLD, which is regarded as the current gold standard in treatment of postoperative lymphedema.

We hypothesized that NP reduces postoperative swelling and pain levels alongside improved functional outcome after TKA as effective as MLD.

## Materials and methods

### Study cohort and randomization

**Fig. 1:**
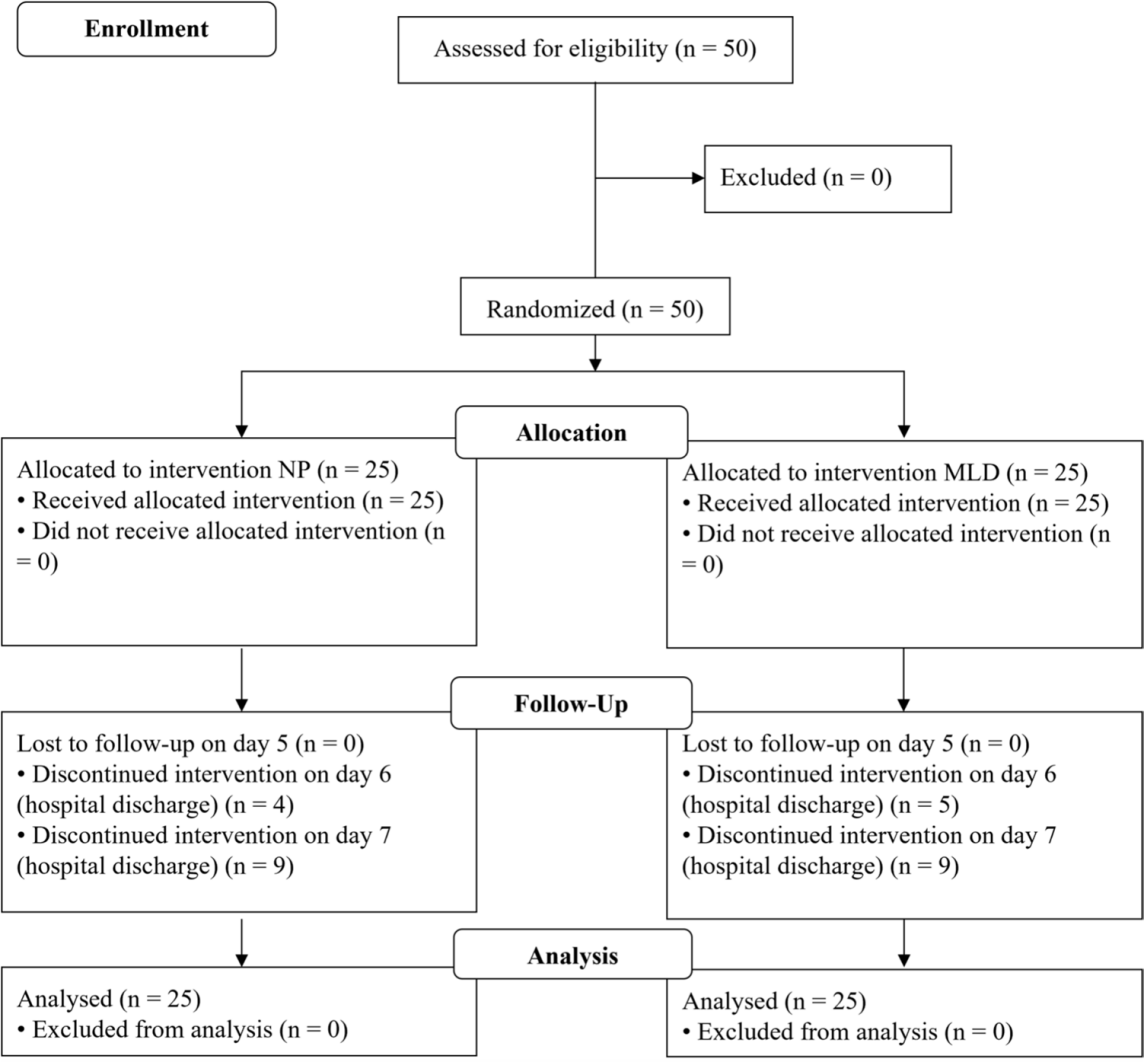
Flow–chart of the study protocol. Reason for lost to follow–up was feasible discharge of patients. No adverse events were documented

A total of 50 consecutive patients undergoing primary TKA were enrolled prospectively. They were divided into two independent groups of 25 subjects each by blinded randomization (Fig. 1). A randomization sequence was generated using free computer software (http://www.randomizer.org), assigning each number from 1 to 50 to either the MLD or NP group. The allocation sequence was prepared in advance by an independent investigator and placed into 50 individual sealed, opaque, and sequentially numbered envelopes. These envelopes were randomly distributed among the included patients immediately prior to the intervention.

Randomization was performed without restriction. The total number of included patients was based on a power analysis, estimated recruitment time, and the requirement that all surgeries were performed by the same surgeon.

Due to the nature of the interventions (manual therapy vs. device-based therapy), blinding of the patients themselves was not feasible. However, outcome assessment was performed in a blinded manner. All staff responsible for measuring the postoperative parameters—such as limb circumference, pain scores and range of motion—were blinded to group allocation. Additionally, the operating surgeon was blinded at the time of surgery. This resulted in a single-blind study design with blinding of the assessor and surgeon.

All TKAs included in this study were performed by the same experienced orthopaedic surgeon using a mechanically aligned technique. A standard medial parapatellar approach was applied in all cases. The decision to implant either a cruciate-retaining (CR) or posterior-stabilized (PS) prosthesis was made intraoperatively, depending on the functional status of the anterior and posterior cruciate ligaments. Soft-tissue adjustments and ligament releases were performed as required, based on intraoperative gap assessment. No constrained implants were used. This consistent surgical protocol ensured minimal variability between patients and allowed for a focused evaluation of the effects of the two postoperative decongestive strategies.

### Inclusion criteria

Patients more than 18 years old with written informed consent undergoing inpatient stay after elective primary TKA were included.

### Exclusion criteria

Exclusion criteria were defined as follows: Acute infection of the lower extremity including wound healing disorders and periprosthetic joint infection (PJI), chronic or acute history of deep vein thrombosis (DVT), chronic or acute history of lymphedema. Subject´s withdrawal from study was able at any time without giving reasons.

### Data collection

Medical data were collected and stored electronically. Pseudonymization was strictly ensured. Primary outcome parameters were defined as the circumference of the affected limb in cm after TKA, secondary outcome parameters were defined as follows: Duration of hospitalization, subjective perception of pain (measured via visual analogue scale, VAS) and affected limb’s ROM. All parameters were examined once preoperatively and on each postoperative day after intervention up to discharge. Measurement of leg circumference was performed at the following 7 anatomical landmarks:

- 20 cm above the knee joint gap
- 10 cm above the knee joint gap
- Knee joint gap
- 15 cm below the knee joint gap
- Ankle
- Foot´s instep
- Ball of forefoot

Knee ROM was measured in maximum extension and flexion using a continuous passive motion (CPM) device to ensure standardized positioning and minimize variability in joint movement assessment. All measurements were performed by the same examiner throughout the study to minimize intra-observer variability. The use of a mechanical device served to limit subjectivity and improve measurement consistency.

All measurements were conducted under consistent clinical and environmental conditions.

### Treatment groups: MLD vs. NP

Treatment of lymphedema started on the first postoperative day after TKA. When performing MLD, therapist started activation of lymphatic tissue around the neck, with the torso and the affected limb following. Using special grip techniques according to the respective body region, the therapist stimulates the absorption of stagnated fluid from the tissue into the lymphatic system. Basic grip techniques are circling, pumping and turning. MLD was performed by trained physical therapist whereas patients were treated by the same therapist in each case.

**Fig. 2:**
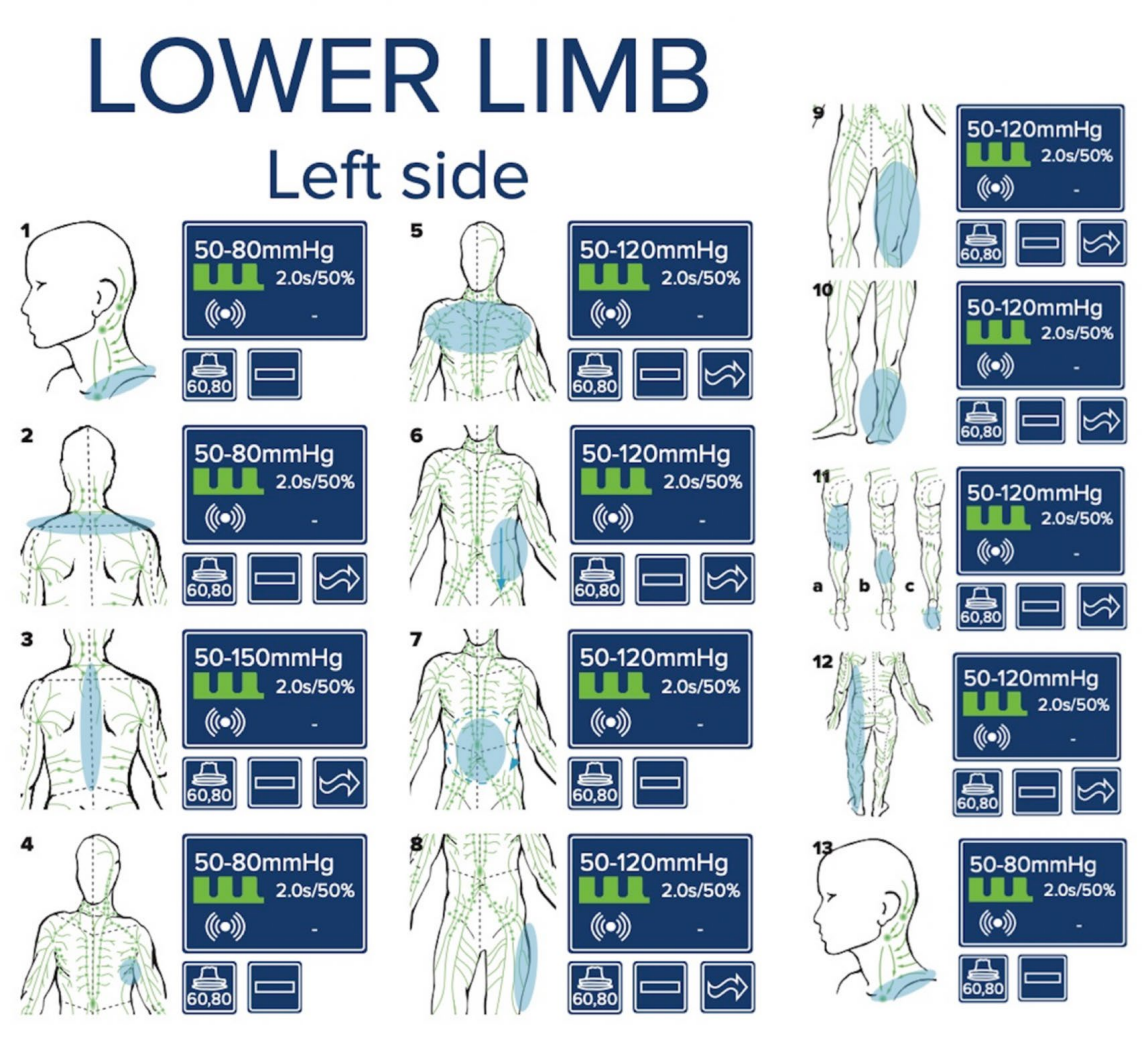
The standardized *Lymphatouch®* treatment protocol is shown. Negative pressure therapy was applied in a defined frequency, pressure and duration

NP is generated in the lymphatic systems of the affected extremity via a silicone-coated applicator (*Lymphatouch®*). It was applied by locally gliding with the “lift and twist” technique. Different pressures (between 20 and 250 mmHg) were applied depending on the skin and soft tissue condition with the frequency being adjusted to 70–90 Hz. During application, pulsed or continuous negative pressure treatment were used, as well as high frequency vibration. NP was performed by same applicant according to a standardized application protocol (Fig. 2). Therapy duration in both groups was 20 min. MLD and NP was performed once every postoperative day. After the treatment, the patient was encouraged to drink 1 L of fluids.

## Statistical analysis

To calculate the required sample size a power analysis was performed with an estimated mean of difference of 2 ± 2 cm (circumferential difference of the affected limb) between groups. This assumption was based on observed empirical data in the management of postoperative swelling after TKA in our clinic. For a power of 0.95 the analysis resulted in 24 patients for each group. 25 patients were enrolled in each group. Student’s t-test was used to test for significant differences between groups in terms of subjects age, body mass index (BMI), affected side and sex.

Student’s t-test was further used to detect differences between outcome parameters. The normal distribution of the test data was assessed with the Kolmogorov–Smirnov test. We used descriptive statistics to summarize the means, standard deviations, range, and confidence intervals (CI). The level of significance was defined as a *p* value of < 0.05.

### Ethics

All procedures performed in our study involving patients were in accordance with the ethical standards of the institutional research committee and with the 1964 Helsinki declaration and its later amendments or comparable ethical standards. Institutional ethics committee approval was given prior to this study (19-1457). The study is registered in the responsible national Clinical Trials Register (DRKS00018837). Presentation of results follows the recommendations of CONSORT (Consolidated Standards of Reporting Trials) [21].

## Results

A total of 50 patients with a mean age of 68.5 years (range: 52–85) were included. Group MLD consisted of 13 female and 12 male patients with a mean age of 66.6 years. Group NP consisted of 11 female and 14 male patients with a mean age of 68.6 years. There were no statistically significant differences between the groups (age: *p* > 0.05; sex: *p* > 0.05). Mean BMI in the MLD group was 31.6 kg/m^2^ and 31.8 kg/m^2^ in the NP group (*p* = 0.904) 16 right and 9 left knees were replaced in the MLD group and 12 right and 13 left in the NP group (*p* > 0.05). No losses or exclusions of patients after randomization were documented. Each patient was available for examination of primary and secondary outcome parameters. Analysis was performed by original assigned groups. Preexisting vascular conditions, such as varicose veins or peripheral vascular disease, were documented based on clinical history. There were no statistically significant differences in vascular status between groups (*p* > 0.05) (Table 1).

**Table 1:** Baseline characteristics of patients in the manual lymphatic drainage (MLD) and negative pressure (NP) groups

Parameter	MLD group (*n* = 25)	NP group (*n* = 25)	*p*-value
Age (years), mean	66.6	68.6	> .05
Sex (female/male)	13/12	11/14	> .05
BMI (kg/m^2^), mean	31.6	31.8	.904
Side of surgery (right/left)	16/9	12/13	> .05
Preoperative VAS (0–10), mean	2.92	4.2	.058
Preoperative ROM (extension/flexion)	0°–4°–104°	0°–4°–107°	.942
Presence of varicose veins (*n*, %)	3 (12%)	4 (16%)	.68
History of vascular disease (*n*, %)	2 (8%)	2 (8%)	1.00

No statistically significant differences were observed between groups regarding demographic variables, preoperative functional parameters, or vascular conditions

### Duration of inpatient setting

Mean hospitalization time was 6.25 days (6.25 days ± 1.7 in NP vs. 6.25 days ± 1.5 in MLD (*p* > 0.05)). After 6 days 21 patients were still included in the NP group and 14 after 7 days. In the MLD group, 20 patients were still present after six days and 15 after seven days.

### Treatment associated outcomes: postoperative swelling

**Fig. 3:**
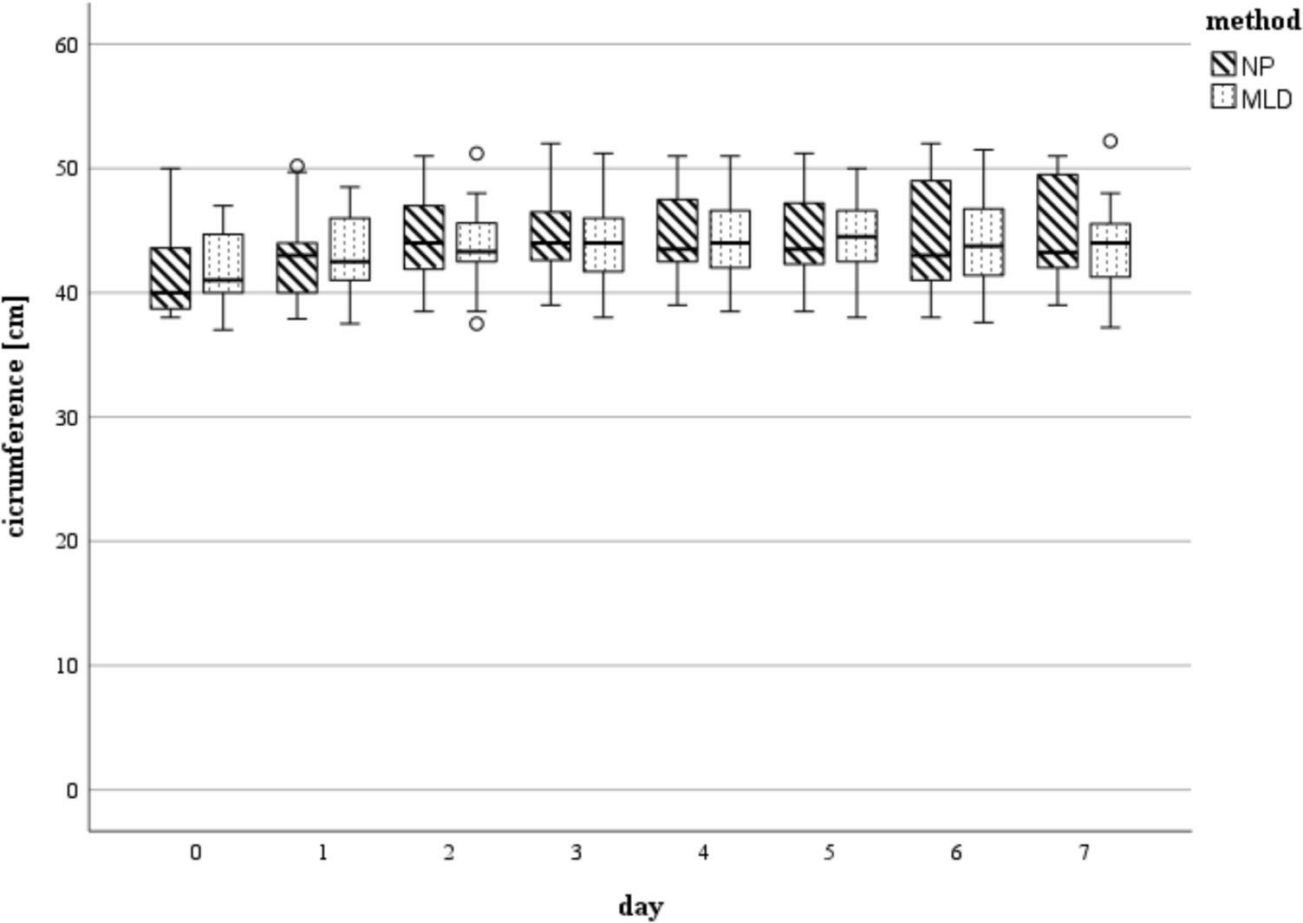
Circumference measured in [cm] at day 0 (preoperative) till day 7 at the level of the knee joint (*n* = 25 each group d0 – d5, *n* = 21 NP, *n* = 20 MLD d6, *n* = 14 NP, *n* = 15 MLD d7): There were no statistically significant differences found between the 2 methods. The horizontal line in the middle of each box indicates the median; the top and bottom borders of the box mark the 75th and 25th percentiles, respectively, the whiskers mark the min and max and the circles mark outliers. *NP* negative pressure, *MLD* manual lymphatic drainage

We witnessed a generalized increase in circumference of the affected limb postoperatively. The maximum swelling was present on the fourth postoperative day with the knee joint being the most affected localization of swelling (plus 6% compared to the status before surgery in both groups). On day 5, swelling decreased continuously. Reduction of swelling after day 5 was equivalent in both treatment groups for the remaining hospitalization without statistically significant differences between MLD and NP (Fig. 3).

### Perception of pain

Mean preoperative pain level in the MLD group was 2.92 compared to 4.2 in the NP group (*p* = 0.058, CI − 0.044 to 2.603). Patients in both groups received analgesic medication according to a modified WHO scheme, which included a basic regimen of Metamizole (2000 mg per day) and Ibuprofen (1800 mg per day). In addition, a sustained-release morphine preparation (maximum 60 mg per day) was available as on-demand medication for both groups.

Actual morphine consumption was assessed and compared between the two groups. The amount of administered morphine was extracted from the electronic medical records for each patient and analysed accordingly. No significant difference in total morphine use was found between the MLD and NP cohorts during the postoperative observation period (*p* > 0.05).

**Fig. 4:**
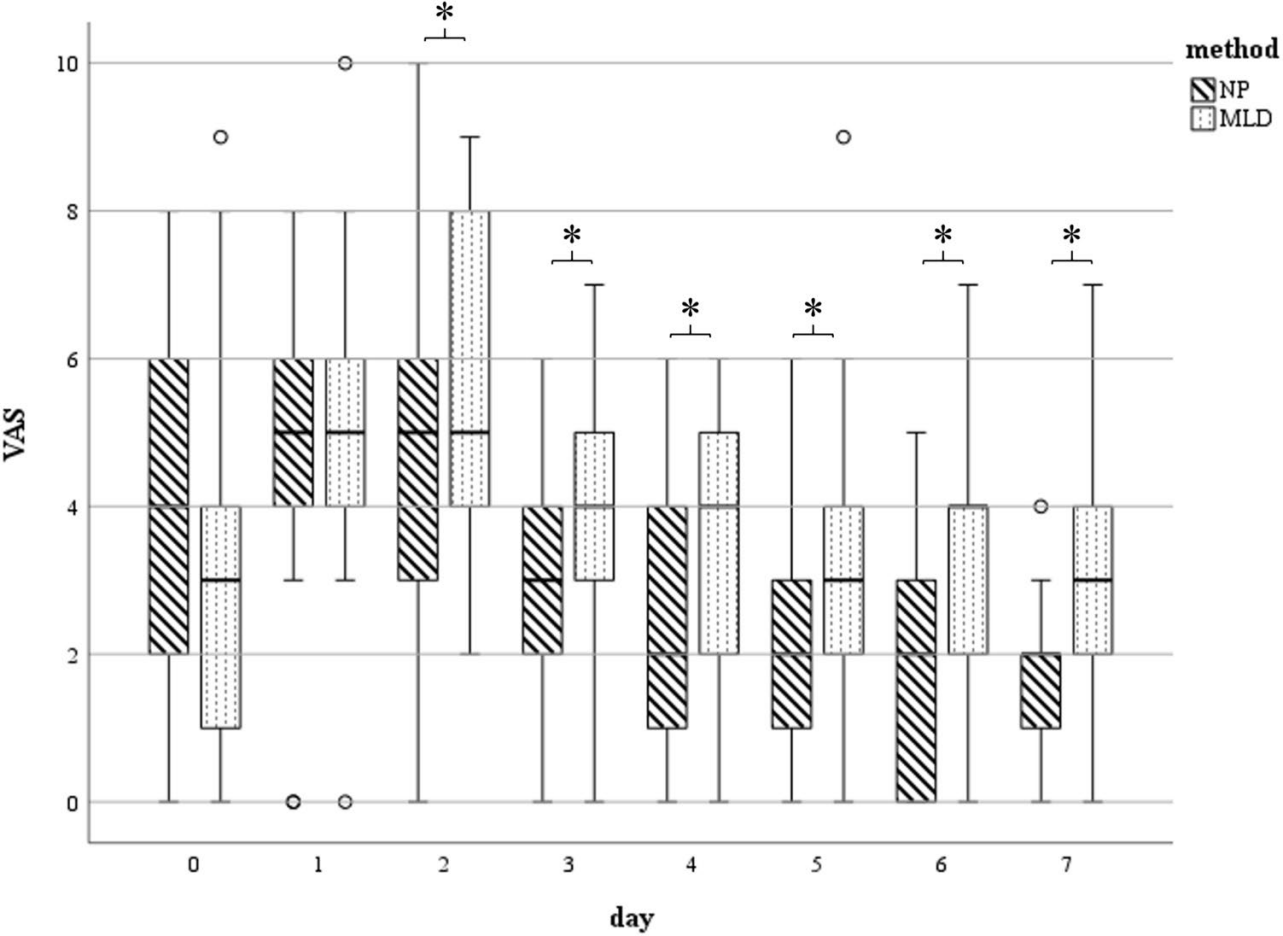
VAS measured at day 0 (preoperative) till day 7 (*n* = 25 each group d0 – d5, *n* = 21 NP, *n* = 20 MLD d6, *n* = 14 NP, *n* = 15 MLD d7): Statistically significant differences (*) between the subgroups were found on days 2–7. The horizontal line in the middle of each box indicates the median; the top and bottom borders of the box mark the 75th and 25th percentiles, respectively, the whiskers mark the min and max and the circles mark outliers. *NP* negative pressure, *MLD* manual lymphatic drainage

Overall pain perception (VAS) was lower in the NP group than in the MLD treatment group. In the NP group, VAS decreased to 1.7 by day 7 postoperatively, corresponding to 40% of the preoperative value. In contrast, patients in the MLD group reported slightly higher pain on day 7 postoperatively compared to their baseline status (3.2 vs. 2.9). It should be noted that patients in the NP cohort had significantly higher baseline pain levels than those in the MLD group (4.2 vs. 2.9). Significant differences in VAS scores between the two cohorts were observed from day 2 to day 7 postoperatively (d2: *p* < 0.05, CI − 2.69 to − 0.42; d3: *p* = 0.033, CI − 1.99 to − 0.08; d4: *p* = 0.041, CI − 1.95 to − 0.04; d5: *p* = 0.012, CI − 2.4 to − 0.3; d6: *p* = 0.014, CI − 2.58 to − 0.3; d7: *p* = 0.29, CI − 2.84 to − 0.16) (Fig. 4).

### Knee range of motion

**Fig. 5:**
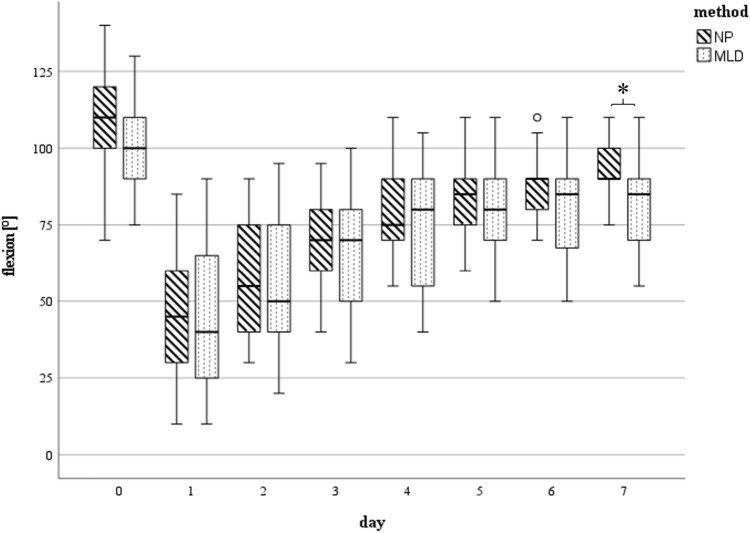
Maximal achievable flexion measured in [°] at day 0 (preoperative) till day 7 (*n* = 25 each group d0 – d5, *n* = 21 NP, *n* = 20 MLD d6, *n* = 14 NP, *n* = 15 MLD d7): Statistically significant differences (*) between the subgroups were found on day 7. The horizontal line in the middle of each box indicates the median; the top and bottom borders of the box mark the 75th and 25th percentiles, respectively, the whiskers mark the min and max and the circles mark outliers. *NP* negative pressure, *MLD* manual lymphatic drainage

**Fig. 6:**
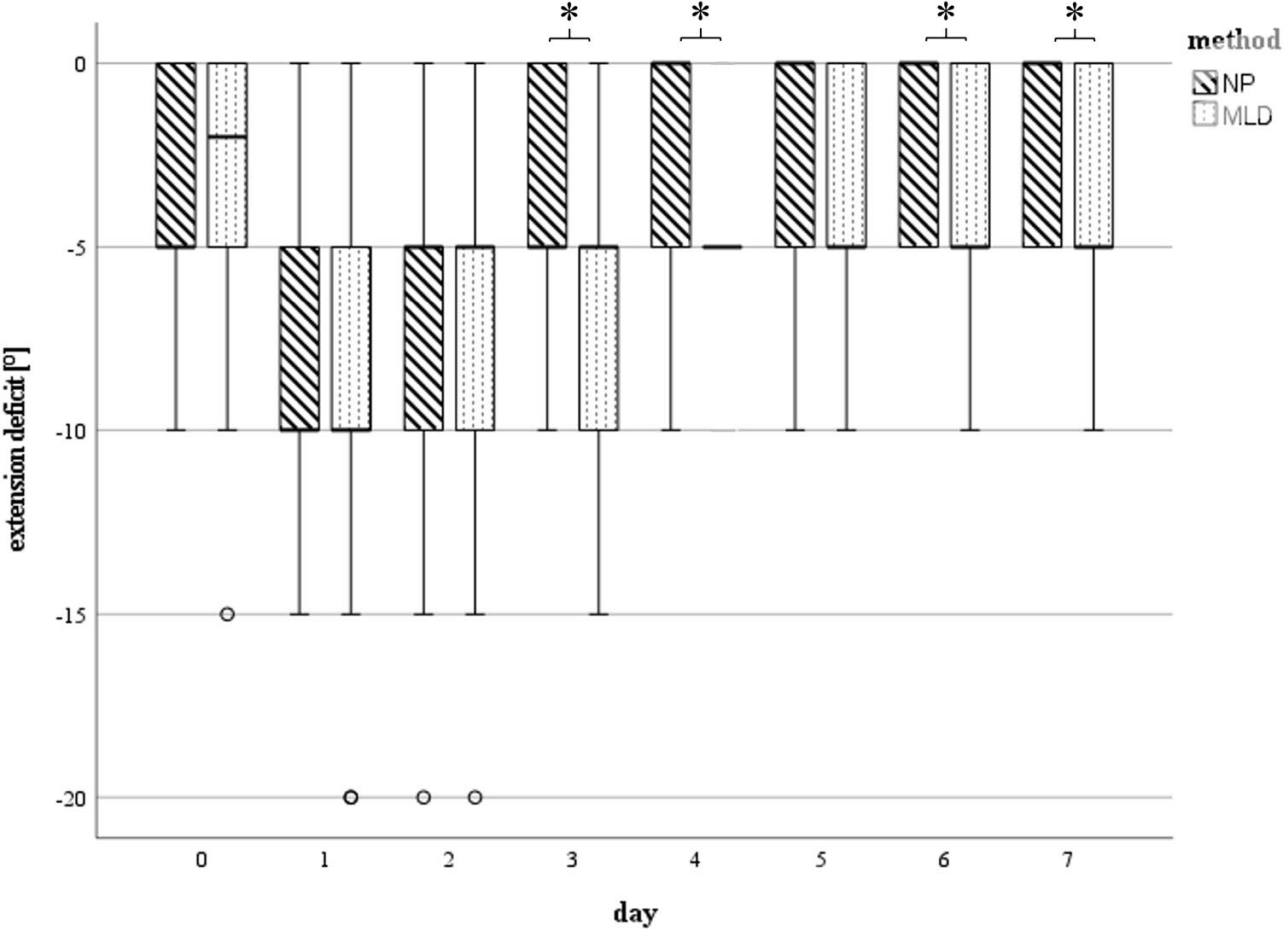
Extension deficit measured in [°] at day 0 (preoperative) till day 7 (*n* = 25 each group d0 – d5, *n* = 21 NP, *n* = 20 MLD d6, *n* = 14 NP, *n* = 15 MLD d7): Statistically significant differences (*) between the subgroups were found on days 3–6. The horizontal line in the middle of each box indicates the median; the top and bottom borders of the box mark the 75th and 25th percentiles, respectively, the whiskers mark the min and max and the circles mark outliers. *NP* negative pressure, *MLD* manual lymphatic drainage

Preoperatively, similar movement function was seen in both groups (mean ROM Extension/Flexion 0°–4°–107° in NP vs. 0°–4°–104° in MLD; *p* = 0.942). Postoperatively, there was a severe limitation of movement but concordantly with reduced swelling of the affected limb, ROM improved constantly during postoperative course. With NP treatment, there was a tendency for better ROM than with MLD. While 7 days after surgery the mean ROM in the NP Group was 0°–2°–92°, a higher restriction of joint mobility was found in MLD group (0°–3°–82°). Statistically significant differences regarding knee joint flexion were documented on day 7 (*p* = 0.26, CI 1.36°–19.63°) (Fig. 4). Significant differences regarding knee extension were found on day 3–7 (d3: *p* = 0.034, CI − 4.61 to − 0.18; d4: *p* = 0.002, CI − 4.79 to − 1.2; d5: *p* = 0.029, CI − 4.15 to − 0.24; d6: *p* = 0.23, CI − 3.86 to − 0.3) (Figs. 5 and 6).

### Adverse events

Both treatment groups showed no adverse events during hospitalization.

## Discussion

In this prospective randomized controlled trial, we compared the effects of a negative pressure application to manual lymphatic drainage in the context to postoperative swelling after primary total knee arthroplasty in 50 consecutive patients.

TKA is considered as a successful surgical procedure for advanced symptomatic knee osteoarthritis. Complications are usually scarce but cannot be entirely avoided. Soft tissue swelling caused by lymphedema is affecting the outcome after TKA and remains a risk factor for early postoperative complications [4, 8, 14]. Increased complication and failure rates in patients undergoing TKA who suffered from extensive postoperative lymphedema could be documented [22, 23]. Current treatments focus on early mobilization, extremity elevation as well as cooling. MLD is regarded as an important treatment option, although scientific evidence is lacking [15, 16]. Optimizing the therapy that reduces limb swelling would improve and fasten patient´s recovery after surgery, while reducing the occurrence of adverse events.

MLD and device-assisted negative pressure treatment was performed on 25 patients each. Limb swelling is at the maximum at 3–5 days after TKA [14]. In this study, maximal limb swelling was documented on day 4 after surgery.

In 2013, Ebert et al. conducted a randomized controlled trial (RCT) to investigate whether it makes any difference at all considering early functional outcome if MLD is performed after TKA [17]. Notably, they could only demonstrate a significant effect on knee joint flexion, while no valid differences in knee joint extension or limb swelling could be shown. In our study, both treatment options (NP and MLD) showed a decrease in limb swelling after TKA.

Furthermore, we were able to confirm previous findings that decongestive measures after TKA have an analgesic effect. Comparing physiotherapy to physiotherapy and additional MLD it could be shown that patients who received additional MLD had significantly lower VAS [17]. An effect that was confirmed in the present study with NP demonstrating superior efficacy regarding postoperative pain levels. Significantly lower pain levels were shown at days 2–7 after surgery.

Our study demonstrated that NP therapy provided comparable results to MLD in reducing lower limb swelling and enhancing mobility recovery following TKA. Notably, NP therapy also led to a significant reduction in pain perception compared to MLD, observed from day 2 post-surgery onward (*p* < 0.05). These findings suggest that NP therapy may offer superior early-stage pain relief, while maintaining equivalent efficacy in controlling postoperative swelling and supporting functional recovery after TKA. Particularly in the immediate postoperative phase, NP demonstrated significant advantages in reducing perceived pain levels compared to MLD, which may contribute to improved patient comfort and earlier mobilization.

The knee joints’ range of motion (ROM) was positively affected by NP as well. Compared to MLD, significant improvements in ROM were observed for flexion on day 7 and for knee extension on days 3 to 7. These results are consistent with previous findings reporting similar ROM values during the first postoperative week following TKA, even with the use of lymphatic drainage techniques [16, 17].

As there was no considerable difference in swelling reduction between the two treatment groups, this result was somewhat unexpected. Typically, joint mobility is assumed to correlate directly with the degree of swelling [24, 25]. While pain reduction may partly explain the improved ROM in the NP group, it is also conceivable that the mechanical effects of negative pressure therapy enhance soft tissue mobility and periarticular tissue dynamics. These effects could facilitate a more physiological restoration of joint kinematics, potentially accounting for the superior early flexion and extension observed. However, specific biomechanical investigations would be necessary to confirm this assumption.

Recent meta-analysis by Lu et al. indicates that the use of MLD following TKA is not without limitations, showing no significant advantage in pain reduction, edema control, or mobility improvement​ [26]. Our findings support this evidence, suggesting that NP therapy is at least equivalent and may be superior in terms of pain reduction.

This study is accompanied by some limitations. We must state, that while both methods showed an equivalent effect on postoperative swelling, we did not include a control group that did not receive any therapy for lymphedema. Moreover, other factors have been reported to affect the functional outcome and ROM after TKA which cannot all be accounted for within the range of this study [27, 28]. Nevertheless, we have minimized confounding variables by demonstrating comparable patient demographics and ensuring that TKA and the postoperative treatment via NP and MLD were performed by the same surgeon or physiotherapist, respectively using an identical implant design in all cases.

Although the trend in many international centres has shifted toward early discharge protocols within enhanced recovery after surgery (ERAS) programs—sometimes even enabling outpatient or same-day discharge following TKA—the present study was conducted in a clinical environment, where extended inpatient care remains standard practice [29]. The mean hospitalization time of approximately six days reflects institutional pathways emphasizing close postoperative monitoring, structured physiotherapy, and safety during the early rehabilitation phase. Nonetheless, it must be acknowledged that prolonged hospitalization may carry its own risks, including increased exposure to hospital-acquired complications. Consequently, further studies are warranted to evaluate the feasibility and effectiveness of NP therapy within ERAS-based or outpatient care settings, particularly regarding its role in reducing swelling and supporting early functional recovery under conditions of shorter inpatient stays.

The potential benefit of NP therapy in the context of healthcare resource limitations—such as the increasing shortage of physical therapists—should be evaluated alongside its economic impact. While device-assisted interventions like LymphaTouch® may reduce manual workload and provide standardized treatment protocols, no cost-effectiveness analysis was conducted as part of this study. Relevant factors such as acquisition costs, maintenance expenses, training requirements, and the need for consumables must be considered when assessing its routine clinical applicability. Moreover, the economic consequences of prolonged inpatient stays—particularly in contrast to established early discharge pathways—should not be overlooked. Given the growing implementation of ERAS protocols and same-day or early discharge following TKA in many healthcare systems, future research should include structured health economic analyses to determine the overall value of NP therapy within both inpatient and outpatient rehabilitation settings.

Of further interest would be a follow-up in outpatient post-treatment phase to examine whether the shown positive effects of NP persist. In the present study, the inpatient acute phase after TKA was investigated.

## Conclusions

This RCT investigated the effect of NP and MLD on limb swelling and its associated consequences after TKA. NP showed an equivalent reduction in limb swelling compared to MLD. In fact, patients' subjective postoperative perception of pain tended to be lower in the NP group. Similarly, ROM tended to be significantly better in the NP than in the MLD group. Relevant differences in patients´ duration of hospitalization could not be demonstrated. To conclude, NP is an effective measure to reduce postoperative swelling and pain and to promote knee mobility.

## Data Availability

No datasets were generated or analysed during the current study.

## Abbreviations

CPM: Continuous passive motion
CR: Cruciate retaining
DVT: Deep vein thrombosis
ERAS: Enhanced recovery after surgery
ROM: Range of motion
MLD: Manual lymphatic drainage
NP: Negative pressure
PJI: Periprosthetic joint infection
PS: Posterior stabilized
RCT: Randomized control trial
TKA: Total knee arthroplasty
VAS: Visual analogue scale
